# Smart About Meds (SAM): a pilot randomized controlled trial of a mobile application to improve medication adherence following hospital discharge

**DOI:** 10.1093/jamiaopen/ooab050

**Published:** 2021-07-31

**Authors:** Bettina Habib, David Buckeridge, Melissa Bustillo, Santiago Nicolas Marquez, Manish Thakur, Thai Tran, Daniala L Weir, Robyn Tamblyn

**Affiliations:** 1 Clinical and Health Informatics Research Group, McGill University, Montreal, Canada; 2 Department of Epidemiology, Biostatistics and Occupational Health, McGill University, Montreal, Canada; 3 Institute of Health Policy, Management and Evaluation, University of Toronto, Toronto, Canada; 4 Department of Medicine, McGill University Health Center, Montreal, Canada

**Keywords:** mobile application, medication adherence, adverse events, pilot randomized controlled trial

## Abstract

**Objective:**

The objectives of this pilot study were (1) to assess the feasibility of a larger evaluation of Smart About Meds (SAM), a patient-centered medication management mobile application, and (2) to evaluate SAM’s potential to improve outcomes of interest, including adherence to medication changes made at hospital discharge and the occurrence of adverse events.

**Materials and Methods:**

We conducted a pilot randomized controlled trial among patients discharged from internal medicine units of an academic health center between June 2019 and March 2020. Block randomization was used to randomize patients to intervention (received access to SAM at discharge) or control (received usual care). Patients were followed for 30 days post-discharge, during which app use was recorded. Pharmacy claims data were used to measure adherence to medication changes made at discharge, and physician billing data were used to identify emergency department visits and hospital readmissions during follow-up.

**Results:**

Forty-nine patients were eligible for inclusion in the study at hospital discharge (23 intervention, 26 control). In the 30 days of post-discharge, 15 (65.2%) intervention patients used the SAM app. During this period, intervention patients adhered to a larger proportion of medication changes (83.7%) than control patients (77.8%), including newly prescribed medications (72.7% vs 61.7%) and dose changes (90.9% vs 81.8%). A smaller proportion of intervention patients (8.7%) were readmitted to hospital during follow-up than control patients (15.4%).

**Conclusion:**

The high uptake of SAM among intervention patients supports the feasibility of a larger trial. Results also suggest that SAM has the potential to enhance adherence to medication changes and reduce the risk of downstream adverse events. This hypothesis needs to be tested in a larger trial.

**Trial registration:**

Clinicaltrials.gov, registration number NCT04676165.

## INTRODUCTION

Prescription medications play an important role in preventing and managing chronic diseases, which impose a significant burden on individuals and health-care systems. To maximize therapeutic benefit, adherence to prescribed medications is necessary. Unfortunately, medication nonadherence is common, with up to 30% of patients failing to fill a new prescription in some cases^1–5^ and 45% failing to take their filled medication as prescribed.^6–10^ Nonadherence is also a problem following discharge from hospital,^11–13^ when several changes are often made to patients’ medication regimens.^14–16^ In a recent study, we found that patients had an average of 4.4 changes made to their medication regimen at discharge, a significant proportion of which were not adhered to: 27% of newly prescribed medications were not filled, 12% of discontinued medications were refilled, and 30% of dose changes were filled at the wrong dose.^11^

The impact of medication nonadherence on health outcomes has been widely documented.^17–19^ Failure to take medications as prescribed increases the risk of adverse events such as emergency department (ED) visits, hospital admissions, and death,^3^^,^^9^^,^^19–23^ as well as associated health-care costs.^24^^,^^25^ Nonadherence to medication changes made at hospital discharge also increases the risk of adverse events,^26^ including the risk of death among patients discharged after a myocardial infarction.^3^ In our recent study, patients who did not adhere to any of the changes made to their medications had a 35% increased risk of adverse events post-discharge compared to patients who adhered to all changes.^26^

Numerous interventions targeting medication nonadherence have been evaluated.^27^ However, only moderate improvements in adherence have been achieved, at best.^28^^,^^29^ Fortunately, digital technologies have emerged in recent years as increasingly popular and potentially powerful tools to provide individualized support to change health behaviors. Various technologies, from telehealth to web-based technologies, have been used to provide a wide range of adherence tools, such as adherence tracking and feedback, patient education and counseling, and medication and refill reminders.^30^^,^^31^ However, many of these interventions involve a limited number of adherence-targeting components. This is despite research that suggests that multicomponent interventions are the most effective,^27^^,^^28^^,^^32^ likely due to the complex, multifactorial nature of nonadherence.^25^^,^^33^

Mobile applications have, by virtue of their versatile nature, the potential to bring several components targeting nonadherence together into one tool. They also have the advantage of being readily available to smartphone owners, who represent 81% of adults in the United States.^34^ Mobile apps have demonstrated success in effecting health behavior change^35^ and their potential to improve medication adherence has also been recognized.^36^ One only has to open the app store on a smartphone to access the dozens of medication-related apps available to users.^37^^,^^38^ However, the majority require tedious manual entry of medications,^38^ few include advanced features beyond pill reminders and adherence tracking,^37–39^ and even fewer have been evaluated.^36^^,^^37^^,^^40^^,^^41^ There is thus a clear need to design mobile apps that integrate multiple, advanced features aimed at improving medication adherence, and to conduct scientific evaluations of their impact on adherence and downstream adverse outcomes such as ED visits and hospital admissions.

We designed and developed Smart About Meds (SAM), a patient-centered mobile application that aims to enhance medication adherence and empowers patients to better manage their medications in accordance with their needs and values. We conducted a pilot randomized controlled trial (RCT) of SAM among patients discharged from 2 internal medicine units of an academic health center. Our objectives were to assess the feasibility of a larger trial and to evaluate the potential of an effect of SAM on outcomes of interest, including adherence to medication changes and adverse event risk.

## MATERIALS AND METHODS

### Study design and context

We conducted a pilot RCT among patients discharged from the 2 internal medicine units of the Royal Victoria and Montreal General sites of the McGill University Health Centre (MUHC). The MUHC is a consortium of 5 tertiary hospitals in Montreal, Quebec, where prescription drug insurance is mandatory and provided by the provincial health insurer (Régie de l’assurance maladie du Québec—RAMQ) to those who are over the age of 65, are not covered by their employer, or are on welfare.

Enrolled patients were randomized using permuted block randomization with varying block sizes of 2 and 4, with equivalent numbers of patients randomized to intervention and control in each block. Patients in the control arm received usual care at discharge, whereas those in the intervention arm received, in addition to usual care, access to the SAM mobile application. Given the nature of the intervention, study participants and recruiting research assistants were not blinded to group allocation, but data analysts were.

Following discharge from the hospital, patients were followed for 30 days, during which app use was recorded in our databases and used to determine utilization rates. Pharmacy claims data were obtained for the 30-day follow-up period to measure primary adherence to medication changes made at hospital discharge, and physician billing data were obtained to identify the occurrence of adverse events (ED visits and readmissions).

### Study population

Patients were eligible for this study if they were 18 years of age or older at the time of hospital admission, were covered by the RAMQ prescription drug insurance program, owned a smartphone or tablet device with an internet connection, were fluent in English or French, and were discharged home. Patients who were not prescribed any medications at discharge, had a prognosis of survival of less than 3 months, were transferred to a non-study unit, or were discharged to a rehabilitation center were excluded from the study.

All eligible patients provided written informed consent prior to being enrolled in the study. Patients who were cognitively impaired or otherwise unable to provide informed consent were enrolled if consent was obtained from a legally authorized representative and if the caregiver responsible for acquiring and administering the patient’s medications agreed to use the SAM app on the patient’s behalf. The study was granted ethics approval by the MUHC Research Ethics Board.

### Data sources

Patients’ discharge prescriptions were obtained from hospital charts. Medications prescribed at discharge, along with their reconciliation status (new, continued, dose change, discontinued), dosage, and special directives, were entered by a trained research assistant into structured data fields in a computerized study administration tool developed by our team.

Pharmacy claims data were obtained from the RAMQ for all study participants for the 3 months prior to hospital admission and the 30 days following hospital discharge. These data included information on medications dispensed to study participants, including the drug identification number (DIN), duration of the prescription, and quantity of pills dispensed. These data were linked using the DIN to data tables from Vigilance Santé, a drug database vendor in Quebec, to obtain further information on the strength of the dispensed medication, format, and typical route of administration.

Physician fee-for-service billing data were obtained from the RAMQ for all services provided to study participants in the 3 months prior to hospital admission and the 30 days post-discharge. These data included the date of the provided service and the service location (eg ED, inpatient ward).

SAM utilization data were retrieved from our databases, which store records of every action conducted by patient and caregiver users in the SAM app. Each record is timestamped, linked to a user, and categorized by the type of feature accessed (eg “drug information leaflet”, “side-effect checker”, “message pharmacist”, “resolve adherence alert”).

### Intervention

#### Control arm

Patients in the control arm received usual care at discharge. Following medication reconciliation, they were provided with a written discharge prescription to be filled at their community pharmacy. Patients may or may not have received written or verbal instructions about their discharge prescription or about changes made to their medications.

#### Intervention arm

Patients in the intervention arm received, in addition to usual care, training in and access to the SAM mobile app at discharge. Patients’ own medications were used during training, which consisted of showing patient and caregiver users how to log into SAM and how to access its various features.

SAM was designed based on the Information-Motivation-Behavioral Skills (IMB) theoretical framework, which integrates key concepts from classic health behavior models (Theory of Planned Behavior, Self-Determination Theory, and Health Belief Model) to advance interventions targeting behavior change.^42^ In brief, IMB posits that successful behavior change requires individualized interventions to address the informational, motivational, and behavioral skills needs of patients. For medication adherence, individuals must first understand their condition and how prescribed medications help manage symptoms (information), must be committed to adhering to recommended therapy (motivation), and have the skills and support that facilitate long-term adherence (behavioral skills) (Table 1). 

**Table 1.** SAM features

*Information* **Pill images:** Once a medication is dispensed, an image of the purchased pill is retrieved from Vigilance databases using the DIN and the image is displayed in the patient’s medication list. This information is intended to help ensure that patients take the correct medications at the correct times and as prescribed. **Drug information:** Patient-friendly monographs are provided to help users understand the indications for treatment, as well as the harms and benefits of their medications. Improved awareness about the role medications play in managing health conditions is essential to reducing ambivalence or resistance to continued use.
*Motivation* **Adherence alerts:** SAM integrates data on dispensed medications with the patient’s discharge prescription and uses decision algorithms to alert users to instances in which patients have not been adherent to their discharge prescription. This includes when they have failed to fill prescribed medications, have refilled discontinued medications, or have filled prescribed medications at the incorrect daily dose. In response to an alert, users can select from a dropdown menu an option explaining their situation (eg I did not purchase this medication because I am concerned about side effects). This information is transmitted to the pharmacist managing the patient, who can follow-up with the patient or caregiver to resolve the issue. **Side effect checker:** Patients often discontinue medications due to fear of or prior experience with side effects.^43^^,^^44^ However, when taking multiple medications, it is not obvious which drug, if any, may be contributing to new symptoms. The side effect checker aims to help patients understand which medications may be implicated in a new symptom and prevent discontinuation of medications that are not implicated. Users can look up the side effects of each of the patient’s medications, as well as the frequency of their occurrence. Alternatively, users can access a list of side effects associated with the patient’s overall medication profile, along with the medications in their list that are associated with each side effect. **Interaction checker:** Drug-drug interactions are among the top medication-related informational needs identified by patients.^45^ Providing patients with this information aims to help avoid known interactions, and could also have the benefit of discouraging medication discontinuation due to fear of interactions. This feature generates a list of drug-drug interactions between medications in the patient’s list and any over-the-counter medications they consider purchasing. The severity of each interaction is also displayed.

*Behavioral skills and support* **Caregiver connect:** Patients can identify caregivers involved in managing their medications and provide them with access to the app. This allows caregivers to provide practical and emotional support and to share ideas to facilitate optimal medication management. **Rate my med:** Patients value opportunities to connect with other patients with similar problems and treatments.^46–48^ This feature increases social support by providing opportunities for patients to share their experience with a medication (eg effectiveness and side effects) and read about the experiences of other users using the same medication. **Pharmacist connect:** This feature aims to empower patients and caregivers to seek relevant information, as needed, for shared decision-making and to receive support for problem-solving, including for adherence problems. Patients and caregivers can communicate with hospital pharmacists using a secured messaging service to ask questions related to their medications, describe concerns and side effects, and resolve medication adherence issues. **Pharmacist dashboard:** The dashboard allows hospital pharmacists to manage a group of patients, receive and respond to questions from patients and caregivers, manage adherence alerts or potential medication-related problems, transmit and manage requests for consultation from treating physicians, and document services. In this pilot, hospital pharmacists were available to respond to patients and caregivers for the first 30 days following hospital discharge. Beyond that point, patients and caregivers still had access to the app, with the exclusion of the pharmacist connect feature.

**Figure 1.**
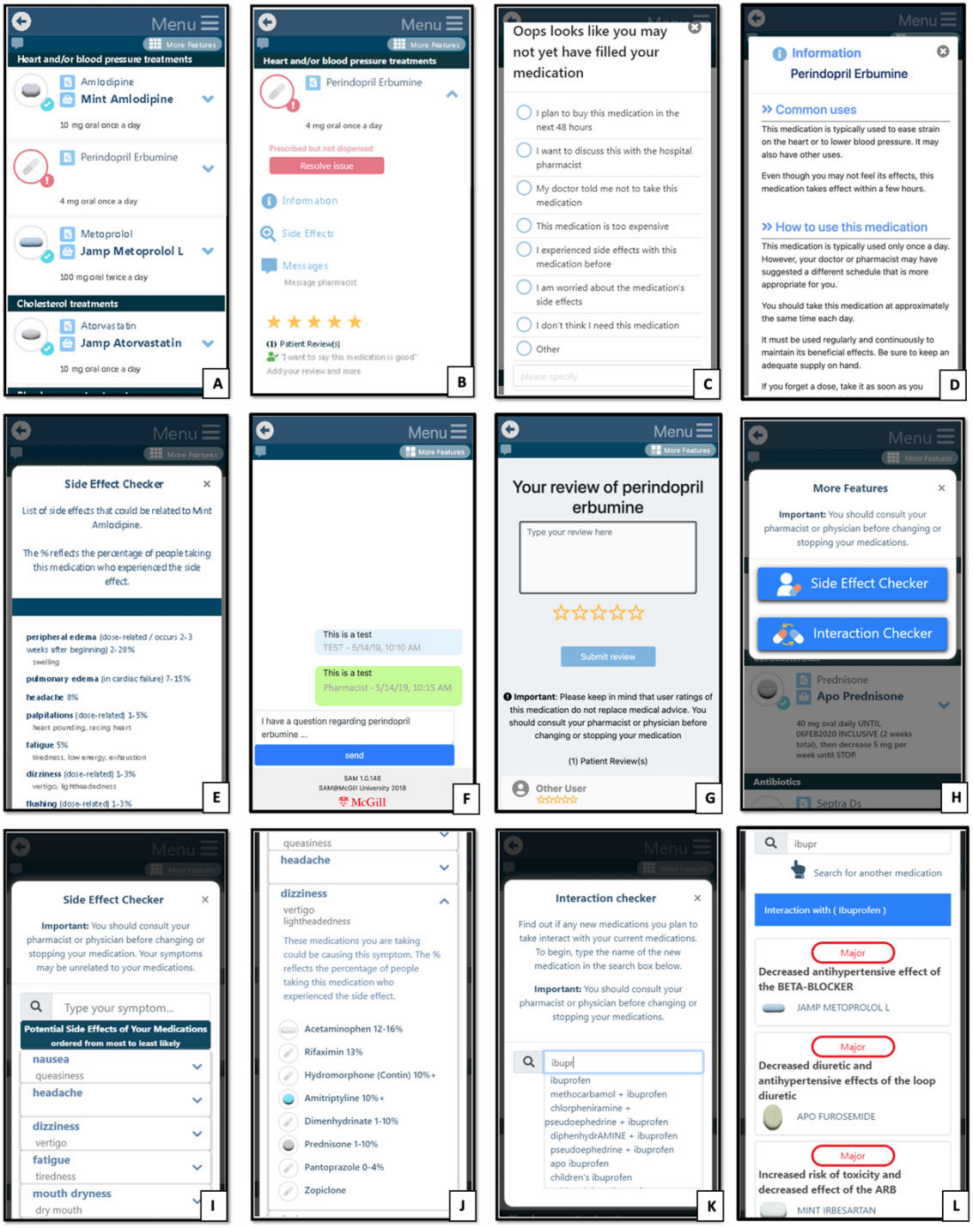
SAM main features. (A) List of prescribed and dispensed medications grouped by therapeutic class, with pill images and prescribed dosage. (B) Dropdown menu of one medication, showing buttons for an adherence alert, drug information leaflet, side effects, messaging feature, and Rate My Med feature. (C) Options to resolve an adherence alert. (D) Patient-friendly drug information leaflet. (E) List of side effects of one medication, with frequency of occurrence. (F) Pharmacist connect feature to send message to pharmacists. (G) Rate My Med feature. (H) Menu to access additional features. (I) Side effect profile of all of the user’s medications. (J) Medications associated with one of the side effects in the user’s side effect profile. (K) Search box for the interaction checker—user searches for medication they consider purchasing. (L) Interaction checker—displays interactions between medication selected in K and the user’s medication profile.

**Figure 2.**
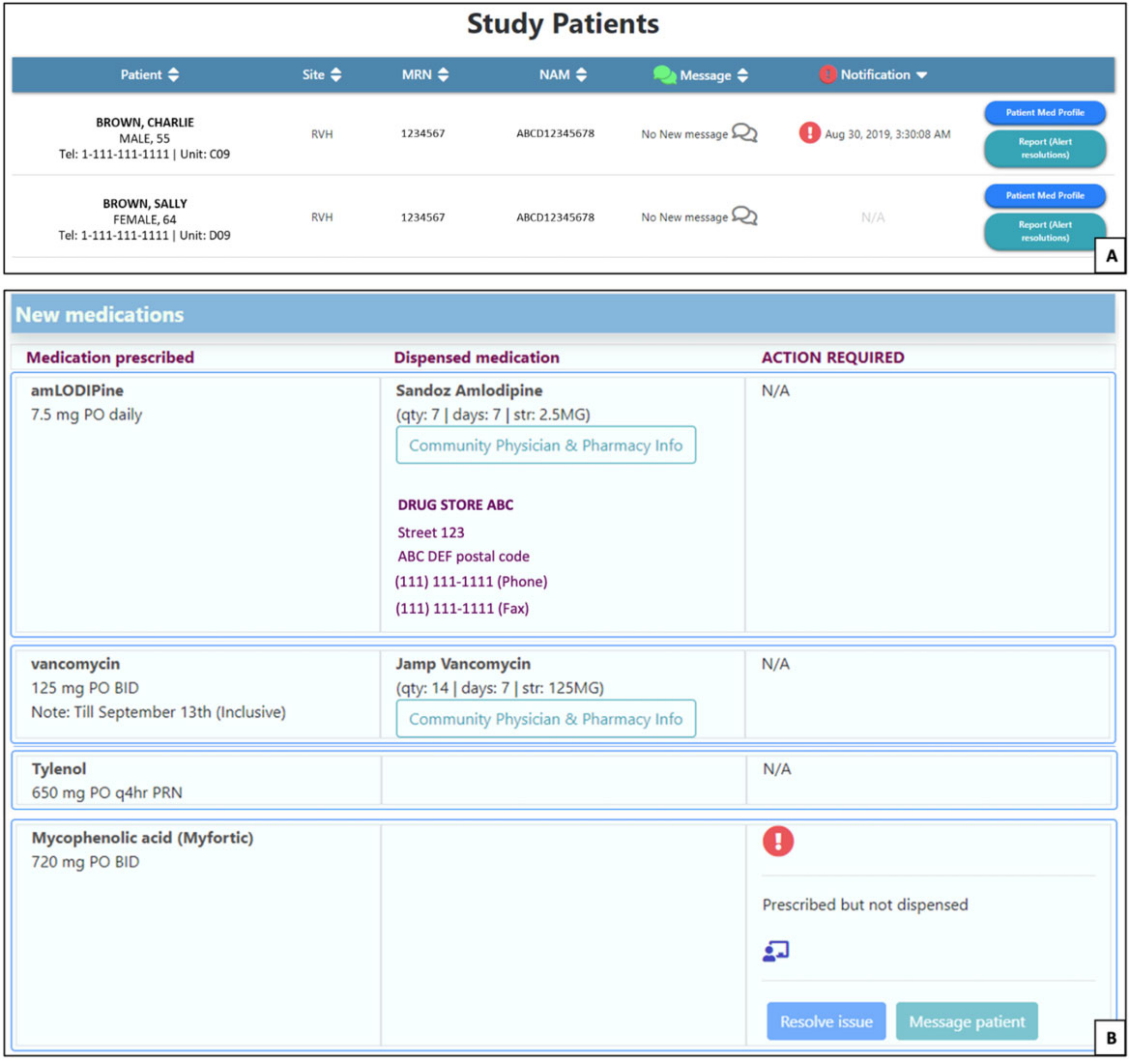
SAM pharmacist dashboard. (A) Main dashboard listing patients using the app who the pharmacist is currently managing. Patient information, hospital discharge site, hospital identification number (MRN), and medicare number (NAM) are displayed. New incoming messages from patients or caregivers are displayed, as are any unresolved notifications (i.e. adherence alerts). Through this dashboard, the pharmacist can also access the patient’s medication profile and documentation services. (B) Patient’s medication profile, with prescribed medications and dosage in the far left column, dispensed medications (if any) in the middle column along with dispensed strength, duration, quantity, and dispensing pharmacy, and any adherence alerts that require attention in the far right column.

SAM was also designed with input from patients and caregivers through a user-centered design development process. The app retrieves the patient’s prescribed medications from the study administration tool and dispensed medications from RAMQ pharmacy claims data. SAM matches dispensed medications to prescribed medications based on ingredient, and generates a patient-friendly list of prescribed and dispensed medications in which medications are grouped by therapeutic class. The app also offers several features aimed not only at addressing barriers to adherence, but also empowering patients to be more informed about their medications and better able to manage them. These include pill images, drug information leaflets, adherence alerts, side effect and interaction checkers, pharmacist and caregiver connect features, and a rate-my-med feature (Table 1, Figures 1 and 2). These features empower patients to actively access information about their medications in a manner consistent with their needs and values, rather than having to depend on a health professional to convey this information.

### Outcome measures

As part of usual care, medication reconciliation was conducted for all patients at discharge. This process, which consists of comparing patients’ community medications to those prescribed at discharge, allows for the assignment of a reconciliation action to each medication prescribed at discharge: new, continue, discontinue, or dose change. New medications are those which patients were not taking prior to hospitalization and which were newly prescribed at discharge. Continued and discontinued medications are those which patients were taking prior to hospitalization and which were re-prescribed as is or discontinued at discharge, respectively. Dose changes are medications that patients were taking prior to hospitalization and that were re-prescribed at discharge, but at a different daily dose. For the purposes of this study, we defined medication changes as medications that had a reconciliation status of new, discontinue, or dose change at discharge.

#### Primary adherence to medication changes

Primary adherence was measured at the medication level as the proportion of medication changes adhered to during the 30-day follow-up period. Adherence was measured overall and by study group, and separately for each type of medication change.

Primary adherence to medication changes was assessed by comparing medications dispensed to patients in the 30 days post-discharge to their discharge prescriptions. Thirty days is a typical length of follow-up for measuring primary adherence, which is usually defined as a new prescription being filled within this timeframe.^49^ In this study, primary adherence to medication *changes* was defined as (1) filling newly prescribed medications within 30 days, (2) not refilling discontinued medications, or (3) filling dose changes at the correct daily dose. For new and discontinued medications, adherence was assessed only for medications reimbursed by the provincial drug formulary and, for new medications, those which were not prescribed on an “as needed” basis. For dose changes, adherence was assessed only for those medications that were actually filled post-discharge. This ensured that our measurement of adherence to dose changes did not include patients who used a leftover supply of medication to modify the daily dose.

The prescribed daily dose was measured by multiplying the prescribed dose per intake by the prescribed number of daily intakes. The dispensed daily dose was measured by multiplying the strength of the dispensed medication by the quantity dispensed and dividing by the duration of the prescription. If the dispensed daily dose of a medication differed from the prescribed daily dose by 25% or more, the medication was considered to have been dispensed at the wrong daily dose. Dose changes dispensed at the incorrect daily dose were considered nonadherence, even if the result of a dispensing error.

#### Adverse events: emergency department visits and hospital readmissions

Adverse event occurrence was measured, overall and by study group, as the proportion of patients who had an ED visit, hospital readmission, or either event in the 30 days post-discharge. Medical fee-for-service billing data were used to identify ED visits and hospital readmissions occurring during follow-up. Previous research has shown that emergency physicians accurately diagnose admissions as medication-related only 51% of the time and of those accurately diagnosed, only up to 28% are reported as medication-related in administrative health data.^50^^,^^51^ Given this low sensitivity of detecting medication-related ED visits and hospital readmissions using administrative health data, we included all such events in the outcome regardless of the recorded diagnosis.

#### SAM app utilization

Time-stamped records of each action conducted in SAM were retrieved from our databases. Overall SAM utilization was measured as the proportion of intervention patients who used SAM post-discharge. Among those users, utilization rates for various SAM features were measured as the median number of times each feature was accessed, per patient and caregiver user, over the 30-day follow-up period.

Records of pharmacist actions in the pharmacist dashboard were similarly obtained to determine the number of times each action was conducted.

### Data analysis

Descriptive statistics were used to characterize the study population (age, sex, caregiver presence, prescribed medications, and prior ED visits and hospitalizations) and to assess adherence to medication changes, the occurrence of adverse events, and SAM app utilization. Medication adherence and adverse event rates in intervention and control groups were assessed using an intention-to-treat approach, that is, patients randomized to the intervention group who did not use the app post-discharge were not excluded. Utilization of SAM features, on the other hand, was assessed only among intervention patients who used the app post-discharge. All analyses were conducted using SAS version 9.4.

## RESULTS

### Study cohort

**Figure 3.**
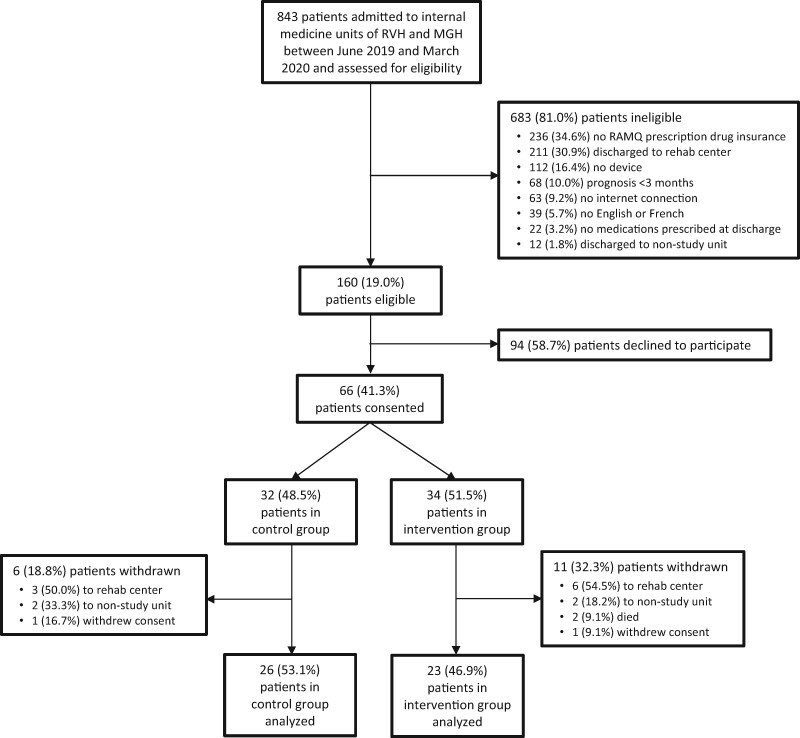
CONSORT flow diagram.

Between June 2019 and March 2020, 843 patients admitted to study sites were assessed for eligibility (Figure 3). Of these, 683 (81.0%) were ineligible for various reasons, chief among which were the lack of RAMQ prescription drug insurance (*N* = 236, 34.6%), expected discharge to a rehabilitation center (*N* = 211, 30.9%), and lack of a smartphone or tablet device (*N* = 112, 16.4%). The remaining 160 (19.0%) patients were eligible, among whom 66 (41.3%) consented to participate and were enrolled in the study. Following randomization, which allocated 32 (48.5%) patients to the control group and 34 (51.5%) to the intervention group, 15 (22.7%) patients became ineligible because of unexpected discharge to a rehabilitation center (*N* = 9, 60.0%), unexpected transfer to a non-study unit (*N* = 4, 26.7%), or death (*N* = 2, 13.3%). An additional 2 patients withdrew consent during follow-up (1 intervention, 1 control). This left 49 patients, 26 (53.1%) in the control group and 23 (46.9%) in the intervention group, who were analyzed. The pilot trial was unexpectedly terminated in March 2020 due to the Coronavirus pandemic.

### Patient characteristics

The majority of study participants were male (*N* = 30, 61.2%) and the median age was 64.6 years (interquartile range [IQR] 45.3–75.0) (Table 2). Participants in the intervention group were younger than those in the control group (median age 54.6 vs 68.6 years, respectively) and less likely to be male (52.2% vs 69.2%, respectively). In the 3 months prior to the index hospitalization, 29 (59.2%) patients had at least one ED visit and 10 (20.4%) were hospitalized. A similar proportion of intervention and control patients had a hospital admission prior to the index hospitalization (21.7% and 19.2%, respectively), but a larger proportion of intervention patients (65.2%) had an ED visit compared to control patients (53.8%). During enrollment, 8 (16.3%) patients indicated that a caregiver aids them with medication management, 1 (4.4%) of whom was in the intervention group and 7 (26.9%) who were in the control group. The caregiver in the intervention group was granted access to the SAM app.

**Table 2.** Patient characteristics

Patient characteristic	Overall (*N* = 49)	Intervention group (*N* = 23)	Control group (*N* = 26)
Sex, male	30 (61.2%)	12 (52.2%)	18 (69.2%)
Age^a^	64.6 (45.3, 75.0)	54.6 (38.2, 74.2)	68.6 (62.1, 76.4)
Patient has caregiver	8 (16.3%)	1 (4.4%)	7 (26.9%)
Medical services in 3 months prior to admission			
ED visit	29 (59.2%)	15 (65.2%)	14 (53.8%)
Hospitalization	10 (20.4%)	5 (21.7%)	5 (19.2%)
Discharge prescription			
# of prescribed medications^a^	15.0 (11.0, 18.0)	13.0 (8.0, 16.0)	15.5 (11.0, 19.0)
# of continued medications^a^	9.0 (5.0, 13.0)	6.0 (4.0, 12.0)	11.5 (6.0, 14.0)
# of medication changes^a^	7.0 (4.0, 10.0)	6.0 (4.0, 11.0)	7.0 (3.0, 9.0)
# of new medications^a^	3.0 (1.0, 5.0)	3.0 (1.0, 5.0)	3.0 (1.0, 5.0)
# of dose changes^a^	1.0 (1.0, 2.0)	1.0 (0.0, 2.0)	1.0 (1.0, 2.0)
# of discontinued medications^a^	2.0 (1.0, 3.0)	2.0 (0.0, 3.0)	2.0 (1.0, 3.0)
# of medications dispensed in 30 days postdischarge^a^^,b^	11.0 (6.0, 16.0)	12.0 (5.0, 15.0)	11.0 (7.0, 17.0)

Abbreviations: ED: Emergency Department.

a Results presented as median (interquartile range).

b If a medication was dispensed more than once in the 30-days post-discharge, it was counted only once.

Study participants were prescribed a median of 15.0 (IQR 11.0–18.0) medications at discharge, with a median of 7.0 (IQR 4.0–10.0) changes made to their medication regimen. Of medication changes, a median 3.0 (IQR 1.0–5.0) were new medications, 1.0 (IQR 1.0–2.0) were dose changes, and 2.0 (IQR 1.0–3.0) were discontinued medications. Intervention patients were prescribed a slightly lower median number of medications at discharge (13.0) compared to control patients (15.5). There was little difference between intervention and control patients in the median number of medication changes per patient (6.0 vs 7.0, respectively), including new medications (3.0 vs 3.0), dose changes (1.0 vs 1.0), and discontinued medications (2.0 vs 2.0).

In the 30 days following hospital discharge, intervention and control patients were dispensed a similar median number of medications (12.0 and 11.0, respectively).

### Adherence to medication changes

**Figure 4.**
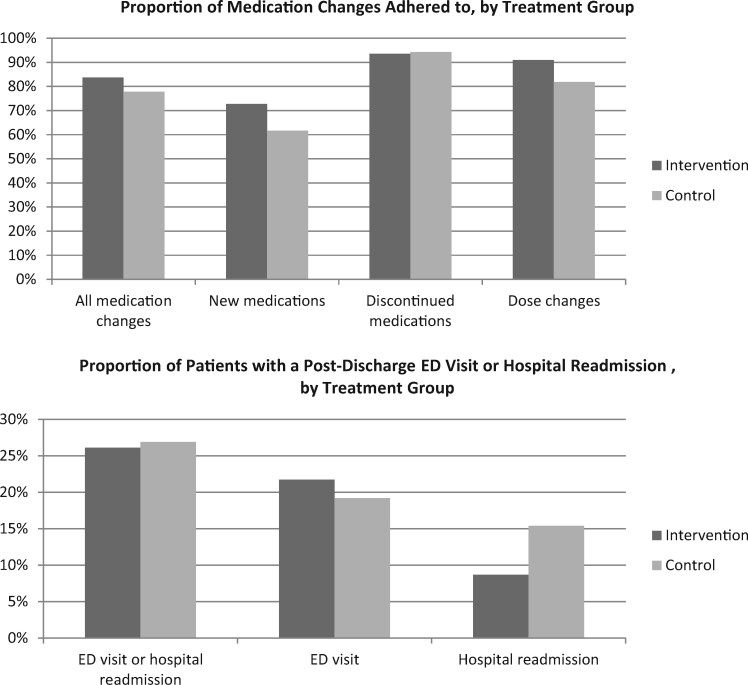
Adherence to medication changes and adverse event rates, by treatment group.

In the 30 days following hospital discharge, a larger proportion of medication changes were adhered to in the intervention group (83.7%) compared to the control group (77.8%) (Table 3, Figure 4). Similar findings were observed for newly prescribed medications (72.7% dispensed in the intervention group vs 61.7% dispensed in the control group) and dose changes (90.9% dispensed at correct daily dose in the intervention group vs 81.8% in the control group). There was little difference between intervention and control groups in the proportion of discontinued medications not refilled (93.5% vs 94.3%, respectively).

**Table 3.** Adherence to medication changes and adverse events in 30 days post-discharge, by treatment group

Proportion of medication changes adhered to, *n*/*N* (%)			
Medication change	Overall	Intervention	Control
All changes	208/258 (80.6%)	103/123 (83.7%)	105/135 (77.8%)
New medications^a^	77/115 (67.0%)	40/55 (72.7%)	37/60 (61.7%)
Discontinued medications^a^	93/99 (93.9%)	43/46 (93.5%)	50/53 (94.3%)
Dose changes^b^	38/44 (86.4%)	20/22 (90.9%)	18/22 (81.8%)

Number of patients (%) experiencing adverse events			

Adverse event	Overall (*N* = 49)	Intervention (*N* = 23)	Control (*N* = 26)

ED visit or hospital readmission	13 (26.5%)	6 (26.1%)	7 (26.9%)
ED visit	10 (20.4%)	5 (21.7%)	5 (19.2%)
Hospital readmission	6 (12.2%)	2 (8.7%)	4 (15.4%)

Abbreviations: ED: Emergency Department.

a Adherence was assessed for new and discontinued medications that are covered by the RAMQ and, in the case of new medications, for those not prescribed “as needed”.

b Adherence was assessed for dose changes that were dispensed in the 30 days following hospital discharge.

### ED visits and hospital readmissions

In the 30 days following hospital discharge, 13 (26.5%) patients had an ED visit or hospital readmission, 6 (26.1%) of whom were in the intervention group, and 7 (26.9%) who were in the control group (Table 3, Figure 4). While there was little difference between treatment groups in the proportion of patients who had an ED visit (21.7% of intervention patients, 19.2% of control patients), a smaller proportion of intervention patients (8.7%) were readmitted to hospital than control patients (15.4%).

### SAM app and pharmacist dashboard utilization

Of the 23 intervention patients, 15 (65.2%) accessed and used the SAM app in the 30 days following hospital discharge (Table 4). Patients who used SAM conducted a median of 16.0 (IQR 8.0–31.0) actions in the app. The side effect checker was the most frequently used feature, having been accessed a median 9.0 times per user. Other frequently used features included the pharmacist connect feature (median 2.0 messages per user) and the drug information leaflets (median 3.0 times per user).

**Table 4.** Utilization of SAM, overall and by feature, and of the pharmacist dashboard

Overall utilization of SAM	
	*n*/*N* (%)
Intervention patients who used SAM	15/23 (65.2%)

Utilization, by feature, among SAM users (*N* = 15)	

SAM feature	Median (IQR) # of times accessed per user

Any feature	16.0 (8.0, 31.0)
Side effect checker	9.0 (2.0, 23.0)
Drug information leaflet	3.0 (1.0, 4.0)
Message pharmacist	2.0 (0.0, 6.0)
Review of medication	1.0 (0.0, 1.0)
Interaction checker	0.0 (0.0, 0.0)
Alert resolution^a^	0.0 (0.0, 1.0)

Utilization of pharmacist dashboard	

Pharmacist dashboard feature	Total # of times accessed

Message patient	86
Alert resolution^b^	66

a Alert resolution refers to the selection, from a dropdown menu, of an option explaining why the patient has not yet filled a prescribed medication, refilled a discontinued medication, or filled a medication at the wrong daily dose.

b Alert resolution refers to the pharmacist’s documentation of the result of follow-up of an adherence alert with a patient.

Over the 30-day post-discharge period, hospital pharmacists conducted a total 152 actions in the pharmacist dashboard (Table 4); 86 of these consisted of sending patient and caregiver users a message through the app and 66 consisted of documenting the results of a follow-up the pharmacist initiated with a SAM user in response to an adherence alert.

## DISCUSSION

In this pilot RCT, SAM was used by the majority (65%) of intervention patients following hospital discharge. An important overlap exists between the 8 intervention patients who never used the app and the 2 who, for logistical reasons, were not trained in the use of SAM. This could explain why these 2 intervention patients did not use SAM. It is also important to note that it is typical for a certain proportion of individuals who download an app to never use it. Although comparable utilization statistics are lacking, one survey of mobile health app use among older adults indicates that 24% of individuals who downloaded a health app never used it.^52^ This could be due to the large number of apps that smartphone users typically download and across which their attention is split. In the survey, 58.9% of individuals who had a health app had at least 11 other apps on their smartphone.^52^ These data support the notion that SAM uptake in this pilot was consistent with typical health app use, which supports the feasibility of future, larger evaluations of SAM. This is despite the relatively low recruitment rate, driven primarily by a low eligibility rate (19%). Our pilot was hindered by other ongoing trials at study sites, which for months restricted our access to patients over the age of 65, who are much more likely to meet the eligibility criterion of possessing public prescription drug insurance. We expect that in a larger trial unhindered by these restrictions, we will be able to recruit study participants at a reasonably faster rate.

Intervention patients who used SAM post-discharge conducted a median of 16 actions in the app, most frequently to check medication side effects, view drug information leaflets, and message hospital pharmacists. Given that SAM is one of very few apps that both display a comprehensive list of medications and have been evaluated, similar data on app use against which we can compare these utilization rates are lacking. However, we can note 2 things. First, our utilization rates do not consider the action of simply viewing the list of prescribed and dispensed medications, as this action was not recorded in our databases. The estimated median of 16 actions per user therefore likely underestimates the actual use of SAM. Second, when interpreting usage rates, it is sometimes more useful to do so in the context of the number of newly prescribed medications, rather than overall medications. This is because patients are more likely to seek information about new medications than those they have been taking for a while. For intervention patients, the median number of new medications at discharge was 3. In this context, a median of 9 actions per user in the side effect checker, for example, seems reasonable.

Our results also suggest that, compared to control patients, intervention patients adhered to a larger proportion of medication changes made at discharge, including newly prescribed medications and dose changes. In addition, although a similar proportion of intervention and control patients had an ED visit post-discharge, a smaller proportion of intervention patients were readmitted to hospital compared to control patients. It is important to note that due to the small sample size of this pilot, no conclusions can be drawn about the effect of SAM on adherence and adverse events. Indeed, this pilot had less than 10% power to detect clinically relevant differences of 5% in adherence rates between treatment groups. However, our results do suggest some potential for SAM to enhance medication adherence and reduce the risk of subsequent adverse events. This hypothesis needs to be tested in a larger RCT.

Larger evaluations may in fact reveal a larger impact of SAM on adherence to new medications and dose changes than what the results of this pilot suggest. This is owing to caregivers’ involvement in medication management, which is known to contribute to medication adherence.^53^^,^^54^ In this pilot, a much larger proportion of control patients (26.9%) had a caregiver compared to intervention patients (4.4%). This imbalance may have underestimated the difference in adherence between treatment groups, a bias that would be corrected in a larger RCT. Other potential biases which would also be corrected in a larger evaluation are those resulting from imbalances in age, sex, and the number of medications prescribed at discharge. Intervention patients were younger than control patients, more likely to be female, and had fewer medications prescribed at discharge. This is important to note, as older patients tend to adhere better to medications than younger patients, as do men compared to women, and those who have fewer prescribed medications.^5^^,^^11^^,^^55^

Interestingly, a similar proportion of discontinued medications was adhered to in intervention and control groups. One possible explanation for this is that, unlike new medications and dose changes, discontinued medications are not displayed in the app unless they are dispensed, triggering an adherence alert. SAM therefore has the potential to improve adherence to discontinued medications, but perhaps only after they have actually been dispensed. The primary adherence measures used in this study consider a patient who had a discontinued drug dispensed as nonadherent even if they did heed the SAM alert and refrained from taking the drug. This could explain why our results suggest no effect of SAM on discontinued medications. On the other hand, secondary adherence measures, which assess continued adherence, may be more likely to show an effect, if it exists. To enhance SAM’s potential for such an effect, we will develop a feature that shows patients the changes that were made to their medication regimen, including which medications were discontinued, which were modified, and which were newly prescribed.

SAM is not the first mobile application to target medication management, but it is one of very few that have been evaluated. Despite hundreds of previously developed medication management mobile apps, a systematic review published in 2020 by Armitage et al^56^ shows only 9 such apps have been evaluated in RCTs for success in improving adherence. Although some demonstrated success, SAM has important advantages over this small number of previously evaluated apps. First, SAM is the first to benefit from real-time linkage to pharmacy claims data. This not only allows for the generation of real-time adherence alerts, which itself is novel, but also allows for an objective measurement of adherence. This is in contrast to self-reported measures used in most previous RCTs,^56^ which overestimated adherence.^57^^,^^58^ In addition, SAM appears to be one of few apps that integrates several advanced features targeting barriers to adherence. Some of the apps reviewed by Armitage et al appear to be basic medication reminder apps.^59–61^ Others offer additional features such as telephone support,^62^ adherence tracking,^63^^,^^64^ refill reminders,^64^ peer support,^63^ symptom tracking,^65^ or even advanced AI monitoring.^66^ However, medication nonadherence is a complex phenomenon arising from a multitude of factors and, unlike SAM, none of these apps appear to take full advantage of the versatility of this medium to target more than 2 or 3 barriers to adherence. Communication with a health-care professional is one example of a feature available in SAM that is a missed opportunity in many others, particularly as it plays an important role in supporting adherence^67–69^ and is valued by patients and caregivers. Finally, unlike most other apps, SAM generates medication lists based on digitized prescription and dispensation data, rather than relying on tedious manual entry of medications by users. This addresses a gap highlighted in a 2016 study that assessed the quality of adherence apps.^70^ The authors underlined the need for apps that interface with prescribing or dispensing data sources to generate a medication list, particularly as older users with complex medication regimens represent those most likely to use and benefit from adherence apps.^70^^,^^71^

Most RCTs reviewed in Armitage et al were characterized by small to moderate sample sizes that had limited statistical power to detect improvements in adherence, much less in the risk of downstream health outcomes. To address this gap in current knowledge, we will build on this pilot and conduct large RCTs of SAM among patients discharged from hospital and patients prescribed medications in primary care settings. The latter will be achieved by linking SAM to an electronic prescriber previously developed by our team,^72^ work which is already in progress. In addition, we will develop a more extensive version of SAM to be tested in these trials. Version 2.0 will include new features such as daily pill reminders and a weekly dosing schedule. We will also incorporate patient preferences into the app via a feature that shows patients alternatives to their medications based on personal preferences regarding medication benefits, adverse effects, and cost. Research has shown that patients differ in their preferences regarding these factors^73–83^ and that tailoring mobile apps to individual users enhances their success in improving medication adherence.^56^

Our pilot had some limitations that should be considered. First, secondary adherence was not measured. This could have overestimated adherence to newly prescribed medications, as patients may not have necessarily taken purchased medications as prescribed.^6–10^ However, adherence is likely to have been overestimated in both treatment groups. Furthermore, we plan to incorporate secondary adherence measures in future evaluations of SAM. This will be done using daily adherence tracking, a new feature that will be developed in SAM, and by obtaining pharmacy claims data over a 6-month follow-up period to measure the Medication Possession Ratio.^49^ Second, patients randomized to the intervention received training in the use of SAM using their own medications. This potential Hawthorne effect, resulting from the additional focus on medications this group received compared to control patients, could have increased primary adherence among intervention patients and led to an overestimated effect of SAM on adherence. However, we expect this bias to be minimal, particularly as control patients were aware of the purpose and outcomes of the study. A third limitation of this pilot is its potentially limited generalizability, as it was conducted in an older, hospitalized population. However, these individuals represent the patient population with the largest number of prescribed medications, on average, and therefore, at the highest risk of nonadherence and associated adverse outcomes.^84–86^ Targeting this population is likely to have significant impacts. That said, our team is working on accessing drug dispensation data for individuals who do not necessarily have public prescription drug insurance. This would allow us to assess the effectiveness of SAM across age groups. We also plan to conduct a trial of SAM among patients prescribed medications in primary care, which would also enhance the generalizability of future studies. A final limitation, which also impacts the generalizability of our pilot, is that eligibility was restricted to patients who possessed a smartphone or tablet device with an internet connection. However, this “tech-savvy” patient population represents those who are most likely to download and use SAM if it were made publicly available, and among whom the effect of SAM on adherence and adverse events is therefore most relevant.

## CONCLUSION

In this pilot RCT of the SAM mobile application, a high uptake of SAM among intervention patients supports the feasibility of a future, larger trial. Results also suggest that SAM has potential to improve medication adherence and reduce the risk of downstream adverse events. This hypothesis needs to be tested in larger evaluations of SAM.

## FUNDING

This work was supported by the Quebec Ministry of Economy, Science and Innovation (MESI) through the Fonds de soutien à l’innovation en santé et services sociaux (FSISSS)—grant number 2-53.

## AUTHOR CONTRIBUTIONS

All authors contributed to the concept and design of the study and of the SAM mobile application. RT acquired the data necessary for the SAM app and outcome measures. MT and TT developed the mobile app. MB and BH coordinated the clinical trial. SM and MB recruited patients. BH conducted statistical analyses and drafted the manuscript. All authors provided feedback on the intellectual content of the manuscript and read and approved the final manuscript.

## ETHICS APPROVAL

This study was authorized by the Research Ethics Board (REB) of the McGill University Health Centre (MUHC)—study number 2019-4597.
